# Supplementary material for: The Systems Biology Research Tool: evolvable open-source software
Source: BMC Syst Biol. 2008 Jun 29;2:55. doi: 10.1186/1752-0509-2-55 (PMC2446383; doi:10.1186/1752-0509-2-55)
Supplement: Additional file 1 — SBRT Archive. An archive of the current version of the Systems Biology Research Tool. [file 1752-0509-2-55-S1.zip › sbrt-1.4.0/doc/developers_guide/api/sbrt/kernel/fba/package-summary.html]

sbrt.kernel.fba


|  |  |  |
| --- | --- | --- |
| **Overview** \| **Package** \| Class \| **Use** \| **Tree** \| **Deprecated** \| **Index** \| **Help** | |  |
| **PREV PACKAGE**   **NEXT PACKAGE** | **FRAMES**    **NO FRAMES**     **All Classes** |  |


---

## Package sbrt.kernel.fba

Provides classes and interfaces for creating objects relevant to
Flux Balance Analysis.

**See:**
  
          **Description**

| **Interface Summary** | |
| --- | --- |
| **CatalyzedFluxome** | This interface is used to represent fluxomes with catalyzed reactions. |
| **ChemRxn** | This interface is used to represent simple chemical reactions. |
| **Fluxome** | This interface is used to represent fluxomes for use in Flux Balance Analysis. |
| **IrrevRxnFluxome** | This class is used to represent fluxomes that contain only irreversible reactions. |

| **Class Summary** | |
| --- | --- |
| **AbstractRxn** | This class is a skeleton implementation of ChemicalRxn. |
| **ExchangeRxn** | This class is used to represent exchange reactions in Flux Balance Analysis. |
| **FluxConstraints** | This class is used to group a set of flux constraints used in Flux Balance Analysis. |
| **IrrevRxn** | This class is used to represent irreversible chemical reactions. |
| **IrrevRxnFluxomeV1** | This class is a concrete implementation of IrrevRxnFluxome. |
| **ReversibleRxn** | This class is used to represent reversible chemical reactions. |
| **SinkRxn** | This class is used to represent sink reactions in Flux Balance Analysis. |
| **SourceRxn** | This class is used to represent chemical species sources in Flux Balance Analysis. |

## Package sbrt.kernel.fba Description

Provides classes and interfaces for creating objects relevant to
Flux Balance Analysis.

---


|  |  |  |
| --- | --- | --- |
| **Overview** \| **Package** \| Class \| **Use** \| **Tree** \| **Deprecated** \| **Index** \| **Help** | |  |
| **PREV PACKAGE**   **NEXT PACKAGE** | **FRAMES**    **NO FRAMES**     **All Classes** |  |


---
